# The use of video review to enhance recognition of intubation attempt duration and success in fetal care center delivery room resuscitations

**DOI:** 10.1038/s41372-025-02240-1

**Published:** 2025-02-25

**Authors:** Morgan E. Hill, Jae H. Kim, Stefanie Riddle

**Affiliations:** Division of Neonatology, Perinatal Institute, Department of Pediatrics, Cincinnati Children’s Hospital Medical Center, University of Cincinnati College of Medicine, Cincinnati, OH USA

**Keywords:** Paediatrics, Outcomes research, Health occupations

## Introduction

Fetal care centers (FCCs) offer comprehensive care from prenatal diagnosis to postnatal management and may provide delivery room (DR) management of infants with complex diagnoses. FCC DR resuscitation teams must execute and adapt Neonatal Resuscitation Program (NRP) guidelines, including performing intubation, for these complex neonates. Adaptation includes prompt intubation for infants with congenital diaphragmatic hernia (CDH) and avoidance of prolonged face-mask ventilation for infants with gastroschisis [1]. NRP recommends the steps of intubation be completed within 30 s [1]. The National Emergency Airway Registry for Neonates (NEAR4NEOS) reports 46% first attempt success rate for DR intubations [2]. There was lack of awareness surrounding duration and success of intubation attempts during DR resuscitations in our FCC. Video review (VR) is a useful tool to evaluate compliance with NRP [3]. We sought to utilize VR to determine duration of intubation attempts and first attempt success rate for our FCC DR resuscitation intubations.

## Subjects and methods

### Study design

This was a single center, observational study of video recorded DR resuscitations. A FCC VR program was established in November 2022. Digital recordings are generated for resuscitations in the Infant Resuscitation Room. Recordings include two views of the team, vital sign monitor, and video laryngoscope screen when applicable. Patient consent was obtained prior to use of recordings. Recordings were analyzed and data was collected by MH. All intubations were included. Intubation encounter, course, and attempt were defined as per NEAR4NEOS [2]. Duration of intubation attempt was defined as time from laryngoscope blade entering the mouth until removal of laryngoscope blade. In all cases, intubation success was confirmed by exhaled carbon dioxide detection.

### Statistical analysis

Data description and analyses were conducted with STATA Version 17.0 (College Station, TX). Descriptive statistics included counts, percentages, means, and standard deviations. Chi-square, *t*-test, ANOVA or Fisher’s exact test (as appropriate) were used.

## Results

Ninety deliveries were recorded from November 2022 to February 2024; 58 neonates (64%) required resuscitation; 20 (22%) required intubation. CDH was the most common indication for intubation (*n* = 8); GI anomalies (*n* = 4) and congenital heart disease (*n* = 3) were other common diagnoses. There were 37 total intubation attempts with a 45% first attempt success rate (Table 1). Success rate was significantly higher for direct laryngoscopy (67%) vs. video laryngoscopy (22%; *p* = 0.037). Nurse practitioners performed the majority of intubations, yet were the least successful group, although this was not significant. Intubation attempt duration was significantly longer for unsuccessful attempts (mean 79 s) vs. successful attempts (53 s; *p* = 0.018) and for attempts with video laryngoscopy (76 s) vs. direct laryngoscopy (56 s; *p* = 0.076). CDH intubation encounters (*n* = 8) had a 75% first attempt success rate; CDH intubation attempts (*n* = 14) averaged 71 s in duration.

**Table 1:** Intubation first attempt success rates and duration of intubation attempts.

	First Attempt Success (percent)	*p*-value^a^
**All Intubation Encounters (*n*** = **20)**	**45%**	
*By Course*		**0.037**
Direct Laryngoscopy (*n* = 15)	67%	
Video Laryngoscopy (*n* = 9)	22%	
*By Provider*^b^		0.44
Neonatal Perinatal Medicine Fellow (*n* = 10)	60%	
Nurse Practitioner (*n* = 13)	38%	
Attending Neonatologist (*n* = 2)	100%	
	**Mean Duration of Intubation Attempt (seconds)**	***p*-value**^c^
**All Intubation Attempts (*n*** = **37)**	**64.8**	
Successful Intubation (*n* = 20)	52.9	**0.018**
Unsuccessful Intubation (*n* = 17)	78.8	
Direct Laryngoscopy (*n* = 20)	55.7	**0.076**
Video Laryngoscopy (*n* = 17)	75.5	
Neonatal Perinatal Medicine Fellow (*n* = 15)	75.3	0.132
Nurse Practitioner (*n* = 20)	60.7	
Attending Neonatologist (*n* = 2)	27.5	

^a^*p*-values calculated with Fisher’s exact test.

^b^Definitions of first attempt success for provider: the provider was successful during his/her first attempt of the encounter.

^c^*p*-values calculated from t test and ANOVA.

## Discussion

The focus of neonatal resuscitation is achieving effective ventilation, and intubation may be the critical step to achieve this during a complex resuscitation. In this study, our intubations had a 45% first attempt success rate and average attempt duration of 65 s, exceeding NRP recommendations.

Duration of attempt is an additional piece of information provided by VR that may be critical in efforts to decrease select tracheal intubation associated adverse events: a recent study demonstrated the median time from stopping positive pressure ventilation to desaturation <80% was 35 s [4]. Particularly relevant to the complex FCC population, our CDH intubation attempts were successful but prolonged. While prior studies have reported time from birth to intubation in CDH patients, (ranging from 89 to 120 s,) they do not report the duration of the intubation attempt [5, 6]. Thanks to our VR program, we have recognized a quality improvement opportunity to decrease duration of attempts to meet NRP recommendations.

In addition, we have also recognized a deficit in our video laryngoscopy competency. Our providers were only successful 22% of the time with video laryngoscopy as compared to 67% of the time with direct laryngoscopy, despite increasing evidence that video laryngoscopy has greater first attempt success rates as compared to direct laryngoscopy [2, 7]. Quality improvement efforts are now being undertaken to increase our first attempt success rate with video laryngoscopy. Future efforts will focus on obtaining reliable data regarding tracheal intubation associated adverse events.

In conclusion, our FCC VR program provided critical insight into our intubation competency. The use of VR to become intimately familiar with all aspects of an intubation encounter and highlight where the team is highly performing and where quality improvement and educational efforts should be focused is the major strength of this study. We recommend that FCCs employ VR as a tool to increase awareness of team performance with the goal of improving procedural safety and success for patients.

## Data Availability

De-identified data will be made available upon request after institutional data-sharing agreements are in place.
